# Drinking to ease the burden: a cross-sectional study on trauma, alcohol abuse and psychopathology in a post-conflict context

**DOI:** 10.1186/s12888-016-0905-7

**Published:** 2016-06-24

**Authors:** Verena Ertl, Regina Saile, Frank Neuner, Claudia Catani

**Affiliations:** Clinical Psychology and Psychotherapy, Department of Psychology, Bielefeld University, Universitätsstraße 25, 33615 Bielefeld, Germany; vivo international, Konstanz, Germany; Clinical Psychology and Psychotherapy, Department of Psychology, Bielefeld University, P.O. Box 100131, 33501 Bielefeld, Germany

**Keywords:** Trauma, Addiction, Alcohol, Substance abuse, Self-medication, Depression, PTSD, Mental health, Conflict, War

## Abstract

**Background:**

It is likely that alcohol use and abuse increase during and after violent conflicts. The most prominent explanation of this phenomenon has been referred to as self-medication hypothesis. It predicts that psychotropic substances are consumed to deal with conflict-related psychic strains and trauma. In northern Uganda, a region that has been affected by a devastating civil war and is characterized by high levels of alcohol abuse we examined the associations between war-trauma, childhood maltreatment and problems related to alcohol use. Deducing from the self-medication hypothesis we assumed alcohol consumption moderates the relationship between trauma-exposure and psychopathology.

**Methods:**

A cross-sectional epidemiological survey targeting war-affected families in post-conflict northern Uganda included data of male (*n* = 304) and female (*n* = 365) guardians. We used standardized questionnaires in an interview format to collect data on the guardians’ socio-demography, trauma-exposure, alcohol consumption and symptoms of alcohol abuse, PTSD and depression.

**Results:**

Symptoms of current alcohol use disorders were present in 46 % of the male and 1 % of the female respondents. A multiple regression model revealed the unique contributions of emotional abuse in the families of origin and trauma experienced outside the family-context in the prediction of men’s alcohol-related symptoms. We found that alcohol consumption moderated the dose-effect relationship between trauma-exposure and symptoms of depression and PTSD. Significant interactions indicated that men who reported more alcohol-related problems experienced less increase in symptoms of PTSD and depression with increasing trauma-exposure.

**Conclusions:**

The gradual attenuation of the dose-effect the more alcohol-related problems were reported is consistent with the self-medication hypothesis. Hence, the functionality of alcohol consumption has to be considered when designing and implementing addiction treatment in post-conflict contexts.

## Background

Numerous epidemiological studies worldwide have shown that adversities (like traumatic experiences, childhood maltreatment, loss of guardians or loved ones, displacement, loss of employment, etc.) and mental and physical health, including alcohol-related disorders are associated (e.g., [1–8]). The consistently found positive association between trauma-exposure and psychopathology has been referred to as dose-effect [9–11]. Given the fact that most of the currently documented 46 highly violent conflicts [12] take place in resource-poor countries, risk factors related to poverty and war tend to accumulate, leaving people especially vulnerable for mental health problems during and in the aftermath of hostilities. Northern Uganda is a current example of a post-conflict society trying to rebuild peaceful communal life since hostilities have ended in 2006. Social, economic, educational and health structures are being rebuilt after more than 20 years of conflict and 10 years of displacement into internally displaced persons camps, mainly due to the fear of forced recruitment by the rebel forces [13]. In settings like northern Uganda access to specialized care is complicated by either a lack of means to reach or pay for mental health services or the lack and disruption of service provision, or both [14–16]. Consequently people are left to cope with psychological strains and symptoms themselves. Many succeed in finding functional coping mechanisms. However, other coping attempts may work in the short run but are not functional long-term due to detrimental side-effects for the affected individual and his or her social surrounding. One attempt to cope with psychopathological symptoms as well as stressors and the related negative affective and physiological states is the use of drugs to alleviate or suppress the suffering. This strategy has been named self-medication [17, 18]. Khantzian [17] further proposes that substances are chosen to fit the specific symptoms they are intended to mitigate and are selected in accordance to their psychopharmacological effects (e.g., alcohol to reduce anxiety). When self-medication is successful, the consumption of alcohol is negatively reinforced and may foster the development of dependence.

Much research in this context has focused on traumatic experiences and related symptoms as stressors. Cross-sectional as well as longitudinal studies in western populations report a link between trauma-exposure and substance abuse and suggest that this link can be explained by attempts to attenuate symptoms of PTSD [19–22]. The majority of studies investigating the timely course of symptom development report preceding or simultaneous onset of PTSD, depression or anxiety symptoms in relation to substance abuse [23–26]. Studies following the trajectory of symptoms at several time-points retrospectively and prospectively found a parallel pattern of PTSD symptom severity and alcohol consumption [27] as well as alcohol-related symptoms [28]. Onset and course of both disorders were strongly associated and the authors interpreted their results in support of the self-medication hypothesis. In line with the self-medication hypothesis full mediation of the relationship between traumatic stress and alcohol use disorders by PTSD and depression was found in samples of military veterans [29] and female reservists [30]. Relapsing patients with comorbid PTSD and substance dependence reported drinking in response to negative emotions or physical discomfort as reasons for their first lapse after treatment, whereas patients without PTSD reported relapsing due to cue-based craving [31, 32]. Likewise relapsing veterans with the comorbidity reported more depression, anxiety and PTSD symptoms prior to drinking than veterans with substance use disorders only [33].

However, findings from studies investigating the trauma, alcohol use and psychopathology link were not always consistent with the self-medication hypothesis. For instance some studies following alcoholics after treatment demonstrated that depression or anxiety did not reliably predict relapse to drinking [34, 35]. Moreover many studies cannot rule out the reverse causality that alcohol consumption increases the risk to experience traumatic events (often called the high-risk hypothesis [36]) or the likelihood to develop symptoms of PTSD in the aftermath of trauma-exposure (often called the susceptibility hypothesis [22, 37]). For instance a longitudinal study in US soldiers of the Kosovo peace-keeping mission reported that predeployment alcohol abuse predicted PTSD symptoms after return [38]. However this longitudinal study seemingly supporting the susceptibility hypothesis cannot rule out possible self-medication, since predeployment alcohol abuse may have developed as a consequence of self-medication secondary to earlier stressors (e.g., child maltreatment). Olema et al. [39] examined two generations of northern Ugandans highly affected by war-trauma and child maltreatment and found both factors independently predicting symptoms of PTSD and depression in the young generation, but only child maltreatment remained a significant predictor in the guardian generation. They concluded that the impact of child maltreatment on mental health may outweigh the effects of war-related experiences. Literature integrating child maltreatment as well as war-related and other traumatic experiences in the prediction of alcohol use disorders in a post-conflict context is still lacking. Western studies stress the significance of child maltreatment in relation to the development and maintenance of substance abuse [40–42]. A recent study reported that especially emotional abuse seems to play a crucial role [40].

Another alternative mechanism that may explain the current research-results may be shared genetic vulnerability for trauma-exposure, PTSD (and potentially other psychopathologies) and alcohol abuse. Examining male twin pairs having served during the Vietnam era McLeod et al. [43] concluded that the same genetic factors that were associated with trauma-exposure also influenced PTSD symptoms and alcohol use.

Whereas high rates of alcohol consumption and linkages to traumatic experiences and symptoms of PTSD have especially been investigated in war-exposed military populations there is limited knowledge on the prevalence of alcohol use disorders and possible links to war-trauma and other psychopathologies among conflict-affected civilian populations that are much more heterogeneous [44, 45]. Some of the few studies in conflict-affected civilian populations have found high rates of hazardous alcohol use in northern Ugandan as well as Georgian internally displaced men [46, 47]. Alcohol-related symptoms were in both studies linked to trauma-exposure. Screening measures of possibly relevant other mental health disorders were only included in the Georgian sample, where symptoms of depression but not PTSD were independently linked to hazardous drinking next to trauma-exposure [46].

The current study meant to expand the just emerging literature, assessing the scale, patterns, determinants and consequences of alcohol use disorders in (post)-conflict settings. On basis of the literature we expected high rates of alcohol abuse in our northern Ugandan sample although the conflict and displacement had ended. We hypothesized that apart from war-related and general traumatic experiences chronologically more distant experiences of maltreatment in the family of origin predict current alcohol-related symptoms. In this context we were interested in the specific contributions of different categories of experiences (experienced, witnessed and perpetrated violence as well as physical, emotional, sexual and witnessed maltreatment in the families of origin). Earlier studies stressed the specific impact of emotional abuse in the families of origin and the exertion of atrocities like killing [40, 48, 49]. Additionally we assumed that alcohol consumption and alcohol-related symptoms respectively moderate the dose-effect relationship between trauma-exposure and symptoms of mental health disorders. We expected a decrease of the dose-effect with increasing alcohol consumption (successful self-medication) rather than an increase of the dose-effect with increasing alcohol intake (susceptibility hypothesis [22, 37]).

## Methods

### Sample selection and participants

In 2010 we conducted a cross-sectional epidemiological study on the intergenerational effects of war [50, 51]. We originally recruited an exhaustive sample of 516 second-grade students from two suburban and seven rural communities and additionally assessed their primary male and female guardians using standardized questionnaires. Exclusion criteria were acute psychotic symptoms and obvious mental retardation. Data on alcohol consumption among guardians were collected within the rural communities only. Within the 368 rural families, primary male guardians were absent in 62, and primary female guardians in three cases. Additionally two male guardians did not wish to participate. Characteristics of the resulting sample of 304 male and 365 female participants are summarized in Table 1.

**Table 1:** Sociodemographic information, abduction history, trauma-exposure, symptoms of PTSD and depression by gender

	Males (*n* = 304)	Females (*n* = 365)
Sociodemographic Information		
Age, mean (SD)	41.46 (11.44)	38.37 (11.04)
Marital Status, N (%)		
single/separated/widowed	18 (5.92)	82 (22.47)
married/cohabiting	286 (94.08)	283 (77.53)
Level of Education, N (%)		
no schooling/some primary	207 (68.10)	351 (96.16)
completed primary or higher	97 (31.91)	14 (3.84)
Trauma-Exposure		
Abduction, N (%)	206 (67.76)	146 (40.00)
Abduction longer than one month, N (%)	95 (46.12)	34 (23.29)
Abduction duration, mean (SD)^a^	5.25 (11.15)	4.17 (12.83)
Age at first abduction, mean (SD)	25.66 (10.28)	22.68 (9.68)
Family Violence event-types total, mean (SD)^b^	6.15 (4.23)	5.60 (4.09)
Family Violence event-types physical abuse^c^	2.40 (1.96)	2.09 (1.87)
Family Violence event-types emotional abuse^d^	2.26 (1.61)	2.08 (1.58)
Family Violence event-types sexual abuse^e^	0.04 (0.20)	0.04 (0.25)
Family Violence event-types witnessed^f^	1.46 (1.40)	1.35 (1.36)
Traumatic event-types total, mean (SD)^g^	13.71 (4.41)	10.49 (4.51)
Traumatic event-types experienced^h^	5.66 (2.04)	4.44 (2.14)
Traumatic event-types witnessed^i^	7.27 (2.07)	5.84 (2.43)
Event-types with forced perpetration^j^	0.78 (1.27)	0.21 (0.74)
Psychopathology		
PTSD Symptoms total, mean (SD)^k^	2.81 (4.42)	2.92 (4.75)
Symptoms Intrusions^l^	1.01 (1.61)	1.00 (1.68)
Symptoms Avoidance^m^	0.84 (1.64)	0.85 (1.68)
Symptoms Hyperarousal^l^	0.96 (1.79)	1.06 (1.97)
Depression Symptoms, mean (SD)^n^	1.41 (0.49)	1.80 (0.66)

^a^in months. ^b^score range: 0–30. ^c^score range: 0–12. ^d^score range: 0–7. ^e^score range. 0–4. ^f^score range: 0–7. ^g^score range: 0–28 for males, 0–30 for females. ^h^score range: 0–11 for males, 0–13 for females. ^i^score range: 0–11. ^j^score range: 0–6. ^k^score range: 0–51. ^l^score range: 0–15. ^m^score range: 0–21. ^n^score range: 1–4

### Measures

Luo (local language in northern Uganda) versions of the screening instruments were created applying recommendations for cultural adaption ensuring content, semantic, criterion and conceptual equivalence [52, 53]. Procedures included translation by bilingual local translators, lexical back-translation and blind back-translation. The evaluation of translated items took place in focus groups comprising of bilingual local counselors familiar with concepts of mental health and study participants from the first two (suburban) schools. During the adaptation process special attention was dedicated to comprehensibility, acceptability and cultural relevance of the items. Due to extremely high rates of illiteracy all information was gathered in interview format.

### Sociodemography

Year of birth, gender, marital status, ethnicity, level of education and history of abduction were recorded for each participant.

### Trauma-exposure

#### History of family violence

Aversive events experienced or witnessed in the respondents’ families of origin were assessed by a 30-item event-checklist. For the analyses the events answered positively were summed up per subcategory (physical abuse, emotional abuse, sexual abuse and witnessed violence). Additionally a total score was created including all positive answers. The questionnaire included items taken from two standard checklists for traumatic experiences in childhood, the Early Trauma Inventory [54] and the Childhood Trauma Questionnaire [55] and had previously been used in northern Uganda [39] and in other cross-cultural contexts [7, 56].

#### General and war-related traumatic events

The Violence, War and Abduction Exposure Scale (VWAES) is a 34-item checklist of potentially traumatic events that was developed especially for use in the northern Ugandan context [57, 58]. It consists of 18 general event-types, adapted from the Clinician-Administered PTSD Scale [59], six rebel force specific event-types that capture events related to the rebel army (e.g., “Have you ever been forced to eat human flesh by the rebel forces?”) and six forced perpetration event-types (e.g., “Have you ever been forced to kill someone by the rebel forces?”). The original checklist was shortened by the four event-types related to family violence. Two items applied to women only (e.g., “Have you given birth to a child during captivity?”). We summed up positive answers to obtain subcategory scores as well as a total score.

### Psychopathology

#### Alcohol consumption and symptoms of alcohol use disorders

Alcohol consumption and alcohol-related symptoms were measured implementing the 10-item interview version of the Alcohol Use Disorders Identification Test (AUDIT) [60]. Items one to three of the AUDIT assess frequency and typical quantity of alcohol consumption as well as frequency of heavy drinking. Items four to six determine symptoms of dependence and items seven to ten establish harmful alcohol use. Items one to eight are coded on 5-point Likert-type scales ranging from zero to four with varying anchor descriptions fitting the content of the respective question. Items nine and ten offer only 3 anchors with scoring options zero, two and four. The sumscore of items one through ten is commonly used for score-interpretation. The AUDIT identifies hazardous and harmful alcohol use and possible dependence being consistent with ICD-10 definitions. A score of 8 to 14 has been established as an indicator for hazardous use, a score between 15 and 19 as indicator for harmful drinking and a score of 20 and above as indicating dependent drinking. The AUDIT has been reported to accurately measure risk across gender, age and cultures [61–63] and had already been successfully employed in northern Uganda [47]. Interviewers were trained to use a conversion table designed for the present study to be able to convert typical types and serving sizes of northern Ugandan alcoholic beverages into standard drinks. One standard drink was defined to contain 13 g of pure ethanol. In the present study, internal consistency for the male subsample being subject matter of the present analyses was good (Cronbach’s α = .85).

#### PTSD symptoms

We used the validated Lou version [57] of the Posttraumatic Stress Diagnostic Scale (PDS) [64] to assess symptoms of traumatic stress. Its 17 items reflect the symptoms of PTSD according to the DSM IV [65]. The frequency of present PTSD symptoms in the four weeks prior to the interview are scored on a 4-point Likert-type scale ranging from zero (“not at all or only one time in the past month”) to three (“five or more times a week or almost always”). Scores are summed up to provide measures of overall and subscale symptom severity. In the present study, internal consistency for the male subsample was good (Cronbach’s α = .89).

#### Depression symptoms

Symptoms of depression were assessed with the 15-item depression section (DHSCL) of the Hopkins Symptom Checklist [66]. Answers are coded on a 4-point Likert-type scale ranging from one (“the symptom bothered/distressed me not at all”) to four (“the symptom bothered/distressed me extremely). The DHSCL has been extensively used for the assessment of symptoms of depression across a wide variety of cultures (e.g., [39, 58, 67–69]). We applied the commonly used procedure of summing up the item-scores and dividing them by the number of validly filled items to obtain an overall measure of depression symptoms. Internal consistency of the Luo version of the DHSCL was good (Cronbach’s α = .89) in the validation study [57] as well as in the present male subsample (Cronbach’s α = .87).

### Procedure

Nine local counselors affiliated with the NGO vivo international carried out the screening interviews. Clinical psychologists experienced in cross-cultural research and familiar with the northern Ugandan context provided supervision and training. The interview phase at the rural sites began after ten intensive training days. Since the interviewers already had many years of experience in conducting clinical interviews using several of the screening instruments (i.e., the VWAES, the PDS, the DHSCL) the training focused on measures the local study team was not familiar with. Amongst others [50, 51, 70] these were the event-checklist assessing a history of family violence and the AUDIT. We practiced the application of the questionnaires in supervised role-plays until all counselors were proficient in using the measures. Counselors were always accompanied to the interviews in the communities by a study coordinator and instructed to approach her in case of arising questions or difficulties. Questionnaires were routinely checked for missing items and inconsistencies on site. Before starting data collection male and female guardians of all second-grade pupils received an invitation for a parent meeting at the school via their children. At the meeting the study project and procedure was explained in detail and participants were encouraged to raise questions. If participants were interested, individual appointments were made with a counselor. Before the interview started, the interviewer explained the study again and obtained written informed consent (signature or fingerprints). Participants did not receive any financial or material benefit for participating in the study.

Research was performed in accordance with the Declaration of Helsinki. Ethical approval was obtained from the ethics committee of the German Research Foundation, the ethical committee of Gulu University, Uganda, and the Uganda National Council for Science and Technology in Kampala, Uganda. We also sought approval from local government departments including the District Education Offices of Gulu and Nwoya Districts, from councils at the sub-county and community levels and from the school officials of the selected schools.

### Data analyses

In order to identify possible predictors of alcohol use disorders and psychopathology in this sample of war-affected men we applied multiple regression models. We calculated models for men only, since hazardous consumption of alcohol was evident in only four of the 365 women. To estimate their distinct influence on currently present symptoms of alcohol use disorders we included location, age, relationship status, education, abduction status and different categories of traumatic experiences simultaneously in our first regression model. Subsequently, in order to test the hypothesis that alcohol abuse moderates the relationship between trauma-exposure and psychopathology as stated by the self-medication hypothesis, we entered the term trauma-exposure x alcohol-related symptoms to the models predicting symptoms of depression and PTSD. Variables constituting the interaction term were centered. Due to rigorous and supervised data collection no missing data occurred. Data analyses were carried out with JMP® Version 10.0 [71].

## Results

Table 1 summarizes sociodemographic and clinical information relevant for the exploration of the assumed self-medication model. Men and women alike reported having been exposed to an average of six different events from the family violence spectrum, apart from numerous other experienced or witnessed event-types like war-events, accidents and natural disasters (see Table 1). The rate of abductions by the rebel forces was higher in males (68 %) than in females (40 %). Abductions had happened on average 15 years back for the men and 13 years back for the women. Half of the abducted men were forced to perpetrate violent acts by the rebel forces, with 14 % having been forced to kill somebody. Of the abducted women 26 % reported having been forced to exert violence, whereof 6 % indicated having been forced to kill. Current mean symptom scores of PTSD according to the PDS were 2.81 (SD = 4.42) for the men and 2.92 (SD = 4.75) for women. Women were currently suffering more from depression symptoms than men with a mean symptom score of 1.80 (SD = 0.66) versus 1.41 (SD = 0.49) on the DHSCL.

**Fig. 1:**
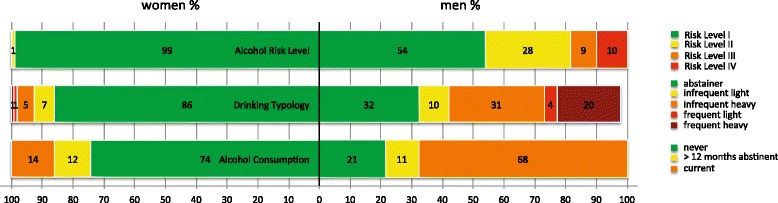
Percentages of men and women categorized according to risk levels as proposed by the AUDIT manual and drinking typology. Drinking Typologies [103] (adapted): abstainer: *never* had a drink or had *none in the past year*; infrequent light drinker: drinking *up to four times a month*, always *less than 5* standard drinks per occasion; frequent light drinker: drinking *two or more times weekly* and *less than 5* standard drinks per occasion. Infrequent heavy drinker: drinking *up to four times a month*, sometimes *6 or more* standard drinks per occasion. Frequent heavy drinker: drinking *two or more times weekly* and *5 or more* standard drinks per occasion. We were not able to categorize 2 % (*n* = 7) of the males since they did not fit in any category. Six men were frequent light drinkers, but had binges of 6 or more standard drinks less than monthly or monthly. One male drank infrequently, but always 5 standard drinks per occasion, he never had binges of 6 or more standard drinks

Concerning consumption of alcohol and alcohol-related symptoms men and women differed greatly. Whereas 74 % of the females reported never having consumed alcoholic beverages in their lives only 21 % of the males reported the same. 27 % of the men indicated currently drinking 2 to 3 times a week or more often (Table 2, Fig. 1). The same frequency of alcohol intake was true for 2 % of the women. Alcohol intake of more than 6 standard drinks on a typical day with consumption were reported by 23 % of the male participants and one woman. Symptoms of current alcohol use disorders applying the AUDIT-threshold-score of ≥ 8 according to Babor et al. [60] were present in 46 % of the male as opposed to 1 % of the female sample.

**Table 2:** Symptoms of alcohol use disorders, frequency and amount of alcohol intake and categorization according to risk levels as proposed by the AUDIT manual [60]

	Males (*n* = 304)	Females (*n* = 365)
Alcohol-related Symptoms, mean (SD)^a^	8.33 (7.69)	0.78 (1.77)
Alcohol consumption, current, N (%)	206 (67.76)	51 (13.97)
Alcohol consumption, but more than one year abstinence, N (%)	33 (10.86)	43 (11.78)
Alcohol consumption, never, N (%)	65 (21.38)	271 (74.25)
Frequency of Alcohol Intake in the past year, N (%)		
never	98 (32.24)	314 (86.03)
monthly or less	53 (17.43)	37 (10.14)
2 to 4 times a month	72 (23.68)	7 (1.92)
2 to 3 times a week	70 (23.03)	6 (1.64)
4 or more times a week	11 (3.62)	1 (0.27)
Alcohol Intake in standard Drinks on a typical day with consumption, N (%)^b^		
currently abstaining	98 (32.24)	314 (86.03)
1 or 2	9 (2.96)	24 (6.58)
3 or 4	75 (24.67)	24 (6.58)
5 or 6	52 (17.11)	2 (0.55)
7 to 9	37 (12.17)	1 (0.27)
10 or more	33 (10.86)	–
Classification according to Risk Level, N (%)		
Risk Level I (AUDIT 0–7)^c^	164 (53.95)	361 (98.90)
Risk Level II (AUDIT 8–15)^d^	84 (27.63)	4 (1.10)
Risk Level III (AUDIT 16–19)^e^	26 (8.55)	–
Risk Level IV (AUDIT 20–40)^f^	30 (9.87)	–

^a^score range: 0–40. ^b^one standard drink is defined as a drink containing 13 g of pure ethanol, e.g., 1 bottle of beer at 330 ml and 5 %, 1 glass of wine (punch) at 140 ml and 12 %, 40 ml of spirits at 40 %. ^c–f^Risk level appropriate intervention proposed in the AUDIT manual: ^c^Education; ^d^Advice; ^e^Advice, Counseling and Monitoring; ^f^Specialist Diagnostics and Treatment

### The effects of different types of trauma-exposure on alcohol-related symptoms

When analyzing the effect sizes of the different types of trauma-exposure and potentially relevant covariates in our simultaneous model (Table 3), having experienced emotional abuse in the family of origin and the amount of general and war-related traumatic event-types experienced contributed most to the level of alcohol-related symptoms.

**Table 3:** Characteristics associated with symptoms of alcohol use disorders

	Alcohol-related symptoms (AUDIT)	
Predictor	β	r
Age	.00	−.05
Currently in a relationship	.07	.05^a^
Completed primary education	−.07	−.05^a^
Abduction	.01	.09^a^
Family Violence event-types physical abuse	.04	.24***
Family Violence event-types emotional abuse	.21*	.26***
Family Violence event-types sexual abuse	.04	.02^b^
Family Violence event-types witnessed	.01	.13^b^
Traumatic event-types experienced	.18*	.23***
Traumatic event-types witnessed	−.09	.15*
Event-types with forced perpetration	.06	.10^b^

Full model’s adjusted *R* ^2^ = .10; *F* (17, 188) = 2.36, *p <* .0027. Location was controlled for entering the model as fixed nominal factor, results are not displayed in the table for reasons of clarity and readability; the location Agweno was significantly associated with the level of alcohol-related symptoms (β = .22*). ^a^Zero-order correlations are represented by point-biserial correlations for dichotomous predictor variables. ^b^Zero-order correlations are represented by Spearman’s ρ. Symbols indicate significance: *p < .05. ***p < .001

### Moderation of the association between traumatic experiences and psychopathology by alcohol-related symptoms

**Fig. 2:**
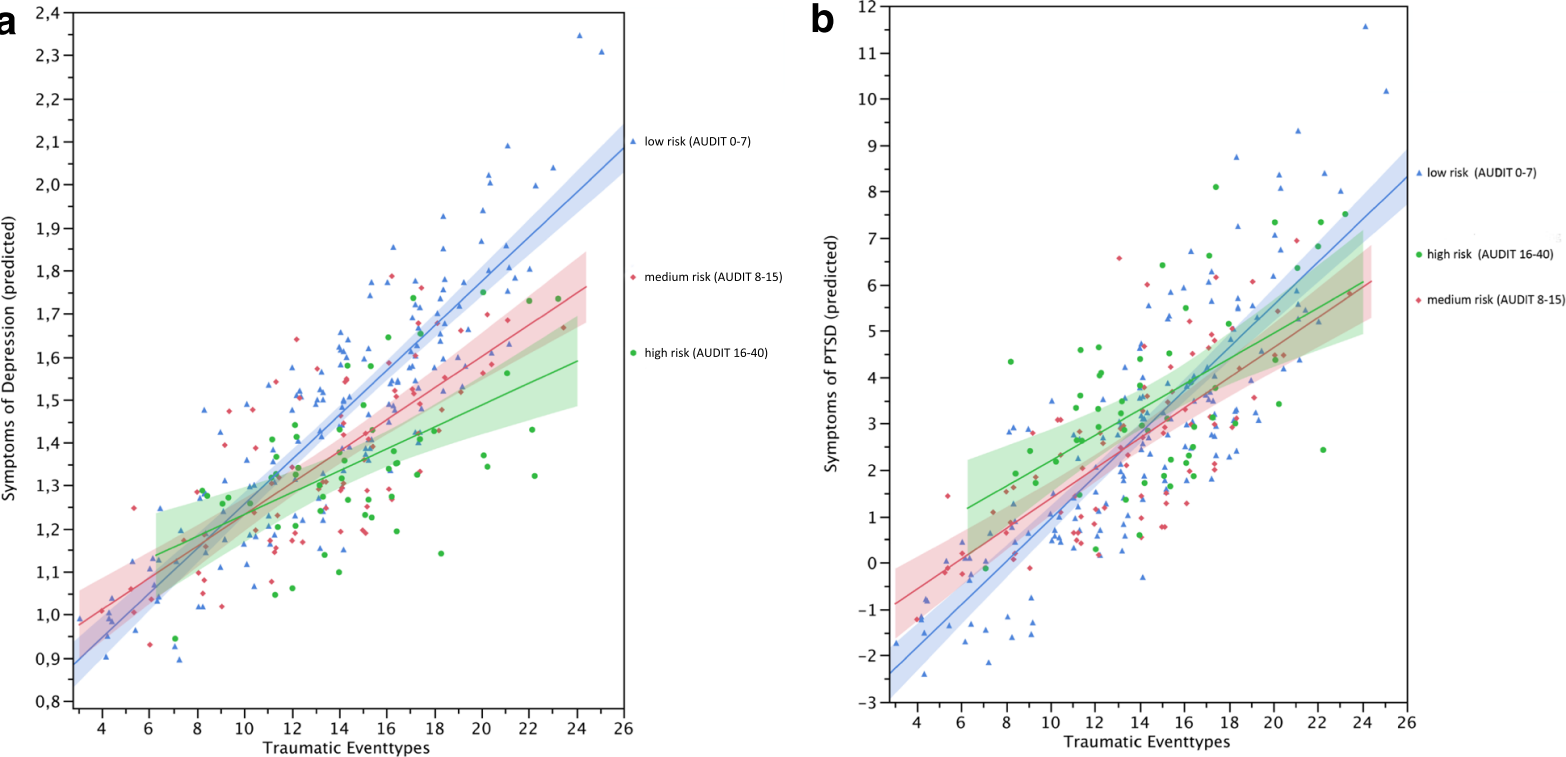
Symptoms of depression (**a**) and PTSD (**b**) as a function of low (Risk Level I), medium (Risk Level II) and high-risk (Risk Levels III and IV) drinking and trauma exposure. For instance, a married, non-abducted man from the community Agweno, who has not completed primary education, is currently abstinent and with his age, trauma-exposure and childhood maltreatment in the family of origin set to the respective sample means has an estimated depression-score of 1.53, which is still below the most frequently used cutoff score for clinically relevant depression of 1.75 [104]. A 10 % increase of reported traumatic event-types for this man is associated with a 5.4 % increase in the estimated depression-score. The same man currently presenting at the threshold of dependent drinking (AUDIT-sumscore = 20) would have a significantly lower estimated depression-symptom-score of 1.37. A 10 % increase of reported traumatic event-types for him would be associated with significantly less increase in the estimated depression-score of 2.8 %

The moderation models shown in Table 4 and graphically depicted in Fig. 2 yielded similar results concerning the prediction of depression and PTSD symptoms. The level of family violence in the family of origin and the level of war-related and general traumatic experiences were the most influential contributors and independently associated with more suffering in both psychopathologies. Moreover, we found a significant interaction of traumatic experiences outside the family-context and alcohol-related symptoms in the models predicting current symptoms of depression and PTSD. Men with more hazardous drinking levels according to the AUDIT (medium and high risk in Fig. 2) showed less increase in depression and PTSD symptoms in response to higher levels of trauma-exposure.

**Table 4:** Moderation of the association between traumatic experiences and psychopathology by alcohol-related symptoms

	Symptoms of depression (*n* = 304)^a^		Symptoms of PTSD (*n* = 304)^b^	
Predictor	β	r	β	r
Age	.05	−.10	−.06	−.14*
Currently in a relationship	−.08	.00^c^	−.04	.03^c^
Completed primary education	.01	.08^c^	.00	.07^c^
Abduction	−.13*	.09^c^	−.01	.16**^c^
Family Violence event-types	.18**	.28***	.24***	.34***
Traumatic event-types	.42***	.39***	.30***	.37***
Alcohol-related symptoms	−.13*	−.13*	.05	.09
Traumatic event-types x Alcohol-related symptoms	−.11*		−.11*	

^a^Full model’s adjusted *R* ^2^ = .22; *F* (14, 289) = 7.18, *p <* .0001. ^b^Full model’s adjusted *R* ^2^ = .23; *F* (14, 289) = 7.62, *p <* .0001. Location was controlled for entering the models as fixed nominal factor, results are not displayed in the table for reasons of clarity and readability; the location Binya (β = −.14*) was significantly associated with depression-symptoms; the locations Idure (β = −.17*) and Binya (β = −.19**) were significantly associated with PTSD-symptoms. ^c^Zero-order correlations are represented by point-biserial correlations for dichotomous predictor variables. Symbols indicate significance: *p < .05. **p < .01. ***p < .001

Exploratory models using the same predictors and splitting the PTSD symptom score up into its clusters reexperiencing, avoidance and hyperarousal indicated that overall prediction was best for symptoms of hyperarousal (Full model’s adjusted *R*^2^ = .22; *F* (14, 289) = 7.14, *p <* .0001) with a significant interaction between trauma-exposure outside the family-context and alcohol-related symptoms (β = −.12*, *p* = 03). The interaction terms concerning symptoms of reexperiencing (β = −.05, *p* = .34) and avoidance (β = −.11, *p* = .06) were not significant.

Using frequency and quantity of alcohol consumption (sumscore of AUDIT items 1 to 3 only) without including symptoms of harmful use and dependence (AUDIT items 4 to 10) as moderator lead to the same results stressing the robustness of our findings.

## Discussion

In the post-conflict society of northern Uganda we found a strong link between trauma-exposure and alcohol consumption in men, resulting in a high prevalence of alcohol use disorders (46 %). Next to war-related traumatic experiences, emotional abuse in the families of origin contributed equally strong to the presence of current alcohol-related symptoms. Supporting the assumption of the self-medication hypothesis, the strong positive relationship between trauma-exposure and symptoms of depression and PTSD (dose-effect) decreased, the more detrimental drinking was reported.

### Prevalence of alcohol-related symptoms and patterns of consumption

We found alarmingly high rates of hazardous (28 %), harmful (9 %) and dependent (10 %) current alcohol abuse in northern Ugandan men, i.e., 46 % ranged above the commonly used AUDIT cutoff score of 8. In contrast only 1 % of the women met this threshold. This finding is consistent with the Global Status Report on Alcohol and Health (2014) [72] that classifies Uganda 5^th^ in the ranking of African countries concerning alcohol per capita consumption confirming high levels of alcohol usage in the whole country. The report stresses the fact that 89 % of the alcohol consumed is unregulated home-brewed and illegally sold alcohol leading to especially high health risks for consumers [72]. Research conducted in northern Uganda when the population was still staying in IDP camps found similar rates of harmful (8 %) and dependent (9 %) drinking and a slightly lower rate (16 %) of hazardous use [47]. A representative study conducted after the northern Ugandan population had left the IDP camps used the Mini-International Neuropsychiatric Interview to assess alcohol dependency and reported alcohol dependency disorder in 7 % of their sample. It is noteworthy that 63 % of this sample was female suggesting that the prevalence rate for males is likely to be considerably higher [73].

### Predictors of alcohol-related symptoms

In our sample emotional maltreatment in childhood was associated with alcohol-related symptoms the same as trauma-exposure outside the family of origin.

Like Roberts et al. [47] we found that a higher number of experienced traumatic event-types was significantly associated with alcohol-related symptomatology. These findings parallel the literature on combat veterans, with the exception that in studies by Maguen et al. [48, 49] committing atrocities (killing) were found to be associated with alcohol-related problems independently from other war-related trauma. In our multiple regression model witnessed traumatic events and forced perpetration of atrocities were not independently related to symptoms of alcohol use disorders. Differences in pre-existing common risk factors for the perpetration of violence and alcohol abuse, such as impulsiveness as well as differences in the need to cope with feelings of guilt may explain the diverging results. Since the men of our sample were forcefully recruited, the exertion of violence may have been carried out after being put under massive pressure (e.g., being held at gunpoint while killing). This may have reduced feelings of guilt related to the perpetration of violence and in turn the need to consume alcohol in order to cope.

Even when taking the more recent war-related and general traumatization into account, emotional abuse in the family of origin was strongly associated with alcohol-related symptoms. In contrast neither physical nor sexual or witnessed maltreatment in the family of origin contributed to the prediction of alcohol-related symptomatology in our multiple model. Cross-sectional and longitudinal studies in western countries have consistently confirmed the detrimental and long-lasting effects of childhood maltreatment on the physical and mental health (including alcohol use disorders) of those affected [41, 74–80]. Literature on the unique significance of emotional maltreatment in relation to other forms of child maltreatment is still scarce and suggests that the impact of emotional abuse may be at least as detrimental [40–42, 81]. Emotional abuse seems to play a crucial role in the development and maintenance of addiction [40]. Whereas a considerable body of literature is emerging investigating the scale, determinants and consequences of childhood maltreatment in (post-)conflict settings [8, 50, 56, 82, 83], there is still a paucity of research examining the effects of both, childhood maltreatment and war-trauma across the lifespan. Nevertheless, the prominent role of child maltreatment next to war-trauma and other traumatic experiences in the prediction of alcohol-related symptoms in the present study seems to be in line with the findings of Olema et al. [39], who have found the same results concerning other pathologies (anxiety, depression).

### Moderation of the dose-effect relationship between trauma-exposure and symptoms of mental health disorders by alcohol abuse

Consistent with the self-medication hypothesis we found that men who reported more alcohol-related problems experienced less increase in symptoms of PTSD and depression with increasing trauma-exposure, indicating a certain functionality of alcohol consumption.

Studies showed that in contrast to their peers without the disorder veterans with PTSD did not profit from alcohol intervention [84] or relapsed earlier [85]. Likewise civilians with a PTSD and substance use disorder comorbidity reported more relapses or unfavorable treatment results [31, 86]. Individuals reporting more traumatic experiences dropped out of inpatient detoxification treatment more readily [87, 88]. All these findings have been interpreted such that traumatized individuals depend more on consumption in order to self-medicate trauma-related negative emotional, physical and cognitive states than non-traumatized patients. Lapses or relapses should be more likely when positive results are perceived or expected in combination with consumption. A positive association of PTSD or depression and alcohol-related symptoms has been a common finding and authors have interpreted these results in favor of the self-medication hypothesis [29, 30, 89–91]. In our study alcohol-related symptoms were not positively associated with symptoms of PTSD and depression. On the contrary, controlling for relevant confounders like sociodemographic variables and maltreatment in childhood we found a negative relationship between alcohol-related symptoms and symptoms of depression. What is more, the significant interactions between trauma-exposure and alcohol-related symptoms in our study imply that the more a person consumed alcohol in our sample, the less adverse impact of trauma-exposure on symptoms of depression and PTSD was evident. These seemingly contradictory results may actually all speak in favor of the self-medication hypothesis. Since our study as well as most of the currently available studies was cross-sectional the key element of timing is lacking in analyses. Assuming that individuals react with alcohol consumption every time when trauma-related mental health symptoms seem overwhelming, we would expect a timely sequence of high symptom load followed by high alcohol intake and a subsequent suppression of trauma-related symptomatology. Thus, when being asked about symptoms within a certain time frame retrospectively respondents may focus either on the exacerbation of symptoms perceived before self-medication or - in case self-medication was successful - on the generally low symptom intensity. Especially when alcohol intake has become a habitual and automatic routine, negative affective or physiological states may not even be consciously noted. When consuming alcohol has become a dominant activity throughout the day and blood alcohol level retains a “therapeutic effect” symptom load may be coherently perceived and rated low. In case of only partly successful or unsuccessful self-medication attempts symptoms of both disorders would be expected to be frequent and intense. Unfortunately the fine fluctuations in symptom load are not captured in most of the current studies including the present study. In sum, the attenuation of the dose-effect in our study may be a correlate of successful self-medication. The fact that other studies rather find a positive relationship between symptoms of PTSD and substance abuse [29, 30, 89–91] may be partly explained by the circumstance that participants in these studies are oftentimes inpatients, patients in rehabilitation or individuals seeking help for either of the two disorders, i.e., self-medication is not experienced as being successful by members of theses groups. In a methodologically superior longitudinal approach Haller and Chassin (2014) [22] intended to shed light on the high-risk, susceptibility, shared vulnerability and self-medication hypotheses focusing on PTSD symptoms. Their results emphasize the importance of considering early life stress, such as child maltreatment when attempting to clarify causal pathways. In their study results would have been supporting the susceptibility hypothesis, stating that adolescent substance abuse is associated with developing PTSD even when trauma-exposure (outside the family of origin) is controlled for. However, taking adversity in the early family environment into account their data supported the self-medication hypothesis.

When we split up the PTSD symptom score into its clusters reexperiencing, avoidance and hyperarousal, the attenuation of the dose-effect was significant for symptoms of hyperarousal only and close to being significant for the avoidance cluster. The Vietnam veterans in Bremner et al.'s [27] study reported that among all symptoms of PTSD alcohol consumption subjectively helped them to control sleep disturbances, nightmares, the constant feeling to be on guard, exaggerated startle responses and the feeling of being cut off from others. The veterans’ self-report data is in line with our results showing a significant moderation, i.e., successful self-medication for the hyperarousal cluster, only.

Nevertheless, especially in the northern Ugandan context another directionality of effect may be possible. Symptoms of depression in our sample may have led to social withdrawal and thus prevented individuals from alcohol consumption, since consumption of alcohol in the Ugandan context is an entirely social phenomenon and drinking mainly occurs in groups. Drinking alone is considered inappropriate. Therefore next to a self-medication effect that may help against symptoms of depression and PTSD exploiting the pharmacological effects of alcohol, the factor of social embeddedness or support due to social drinking may also play a role.

### Limitations

When interpreting our results several limitations have to be borne in mind. Our study design was cross-sectional, i.e., it is not possible to establish causal relationships. The temporal association between psychopathology and alcohol-related symptoms in light of childhood trauma and trauma-exposure outside the family of origin remains speculative. The onset of alcohol use disorders may indeed be secondary to negative emotional, physical and cognitive states or symptoms of mental health disorders that in turn are secondary to trauma-exposure, supporting a self-medication point of view. It is likely that the onset of alcohol abuse and related symptomatology happened secondary to childhood maltreatment in our study. First consumption of alcohol (without necessarily getting drunk) in northern Uganda happens on average at age 15 and an awareness of having an alcohol-related problem develops around age 30 [92], i.e., alcohol-related problems commonly develop after having left the family of origin. However, since fluctuations in frequency and intensity of symptoms and past episodes of mental health disorders were not assessed, we do not know whether alcohol abuse actually did develop simultaneously or secondary to emerging symptoms of PTSD or depression, although the majority of the literature suggests that symptoms of mental health disorders rather precede than follow the onset of substance use disorders [93]. As much as traumatic experiences outside the family of origin and during war and abduction may have influenced alcohol consumption directly or indirectly via psychopathology, it may have been the case that drinking individuals have been more likely to experience traumatic events (high-risk hypothesis) or to develop symptoms of mental health disorders due to their consumption (susceptibility hypothesis). Since the forcefully recruited 68 % of our sample experienced most of the traumatic events outside the family of origin during abduction and alcohol is not available and strictly sanctioned in the rebel army [94] this reverse causality (high-risk and susceptibility hypotheses) is not likely in our study population. Average age at release or escape from the rebel forces was 26 years. For those males that have been abducted onset of alcohol-related symptoms before that age is unlikely.

Moreover, not assessed shared genetic vulnerability is likely to contribute to all psychopathologies investigated in the current study. Unfortunately we did not assess family history of alcohol use or other mental health disorders.

The limitations of our cross-sectional research design to verify the probable existence of successful self-medication could be overcome by longitudinal investigations in community samples in (post)-conflict settings. Additionally more refined methodologies to assess the interplay of negative emotional, physiological and cognitive conditions or symptoms of depression and PTSD and alcohol use, such as ecological momentary assessment could be implemented. Physiological indices [95] could be assessed as objective predictors for motives of consumption. More studies investigating the relationship between trauma, psychopathology and alcohol abuse in non help-seeking populations are needed. Further our findings are limited by self-report and retrospective assessment, i.e., data may be biased by a more or less conscious attempt to use the past to explain and justify current mental health problems and emotional states. Especially self-report concerning the level of alcohol consumption and related problems could have been biased [96]. Finally, our study sample cannot be considered representative. We assessed male guardians from deliberately chosen rural communities, who do have the means to send their children to school. As a result the sample was older than more representative samples in the area [47, 97] and probably more functional, i.e., the scale of mental health problems may rather be underestimated than overrated in the current study.

## Conclusions

In this study we found alarming rates of hazardous, harmful and dependent drinking in northern Ugandan men. Alcohol consumers may be successful in their aim to attenuate or gain control over symptoms and thus report less psychopathology than abstainers in light of severe trauma-exposure. As much as self-medication may have positive effects, the negative effects on the consumer’s health, especially when taking the high ratio of uncontrolled home-brewed alcohol into account, as well as a link of alcohol consumption to the perpetration of intimate partner violence [70, 98–101] and violence against children [7, 50, 83] calls for action. Although the general public and health service providers in East Africa including Uganda are more and more aware of the high rates of alcohol use disorders as well as the related risk of domestic violence and large scale community based prevention programs are being evaluated [102], the factor of alcohol abuse is merely acknowledged as a key factor, but not sufficiently addressed in program development and implementation. Modularized treatment concepts for alcohol use disorders urgently need to be integrated in health service structures offering interventions from detoxification to rehabilitation to prevention. Depending on the level and nature of concomitant problems, like other psychopathology or issues of partner violence or violence against children, short-term interventions could be complemented by modules addressing depression, parenting or non-violent communication. Intervention programs need to be adapted to the respective setting and sound evaluation is warranted.
